# Effects of the COVID-19 Pandemic on University Students' Mental Health: A Literature Review

**DOI:** 10.7759/cureus.54032

**Published:** 2024-02-11

**Authors:** Beatta Zarowski, Demetrios Giokaris, Olga Green

**Affiliations:** 1 Research, Oceania University of Medicine, Pago Pago, WSM; 2 Internal Medicine, Procare Med, Northbrook, USA; 3 Mental Health, Procare Med, Northbrook, USA

**Keywords:** suicidal ideation, depression, anxiety, stress, students, mental health, covid-19

## Abstract

This review aims to focus on the effects of COVID-19 on university students' mental health and deepen our understanding of it. The conclusions are based on the review of 32 studies conducted during the pandemic. This review confirms that university students were at high risk for mental health disorders, heightened stress, and increased sleep comorbidities both pre-pandemic and during the pandemic. This literature review confirmed a few universal trends, i.e., increased stress, anxiety, and depression, during the pandemic. The rates of insomnia, obsessive-compulsive disorder, and suicidal ideation also went up. Overall, female students are at a disadvantage in the development of mental health issues. Male students coped better but may be at higher risk for lethality in suicidal ideation. Students with a history of mental health issues and other comorbidities prior to the pandemic had worse outcomes compared to healthy individuals. The study points to a strong positive correlation between fear and increased rates of stress, anxiety, and insomnia. There is also a positive correlation between declining mental health and online learning. A strong negative correlation was present between physical activity and depressive symptoms. These findings are universal across many countries and regions where the studies occurred.

## Introduction and background

The COVID-19 pandemic began in March 2020 and profoundly impacted university students' mental, emotional, academic, social, and other aspects of life. Mid-pandemic studies [1-5] and pre-pandemic publications, including 2017 WHO mental health estimates, place university students at high risk for mental health dysfunction, with a higher prevalence of psychiatric morbidity compared to the general global population, with 4.4% for anxiety and 3.6% for depression [5]. Unexpected and sudden restrictions, isolation policies, and lockdowns in many countries were meant to protect communities and society at large but had a lot of unintended negative consequences. This review aims to present evidence of the disruptive trends uniquely amplified globally within the university student population and identified in the literature published in 2020-2023 on this topic. The review will analyze 32 peer-reviewed sources focusing on the pandemic's mental, psychological, academic, social, and other effects on university students.

Methodology

This literature review utilized several scientific literature databases, including Science Direct, Google Scholar, Web of Science, and PubMed, to identify articles that include the search criteria, such as those related to the prevalence of anxiety, stress, depression, obsessive-compulsive disorder (OCD), sleep disorders, and suicidal ideation and other symptoms in students in university settings. For cross-reference, the review includes topics identified in scientific literature across different geographical regions and comprises the most prevalent mental health issues. The sources originated from different countries, including the United States, Denmark, UK, Turkey, Saudi Arabia, Serbia, UAE, Italy, Jordan, India, Lebanon, Egypt, Germany, Bangladesh, Iraq, Switzerland, Canada, Australia, and China, offering an international viewpoint on the mental health impact of the pandemic on university students. Each article was published following a scientific peer-review process, ensuring the reliability and validity of the data presented.

Inclusion/exclusion criteria

Specific inclusion and exclusion criteria were put together to preserve focus and relevance. The review included only peer-reviewed literature published between January 2020 and December 2023. The timeframe included research completed during and after the COVID-19 pandemic. Only the publications written in English were studied for inclusion. Studies on the mental health issues and consequences of the COVID-19 epidemic on university students were included, while studies on other populations or unrelated issues were discarded. Inclusion and exclusion criteria are shown in Table 1.

**Table 1:** Inclusion and exclusion criteria for the study.

Criteria	Include	Exclude
Date	Studies conducted during the COVID-19 pandemic between 2020 and 2023	Research before 2020
Topic	Sources related to mental health issues among university students	Other health issues and other populations
Language	Sources in English	Sources not in English
Publication	Peer-reviewed literature, full-text articles, and other sources	Gray literature, conference notes, reports, and abstracts only

## Review

Stress and anxiety

The WHO estimates that the most prevalent mental health disorder is anxiety, affecting up to 1/3 of adults in their lifetime [4]. Following the implementation of quarantines, lockdowns, and suspension of face-to-face teaching, universities and colleges switched to online remote learning modes. This sudden change altered students' functioning, primarily evidenced by increased stress and anxiety. The review demonstrated several trends directly resulting from the COVID-19 pandemic, such as fear of contamination, a rise in OCD, a decline in personal interactions, and long hours pursuing online learning. The review also identified several indirect stressors caused by COVID-19: financial hardships, decreased sleep quality, rise in pre-existing anxiety, depression, and stress. According to pre-pandemic data, direct and indirect stressors have contributed significantly to an overall spike in stress and anxiety. Female gender emerged as an additional factor for increased symptoms and comorbidities during COVID-19.

The most apparent stressor was the danger of contamination [3,6-10]. In the first few months of the pandemic, the university academic population in Turkey cited danger and fear of infection as the most significant stressors affecting their day-to-day lives [3]. American students echoed the same fear of infection among their family members and themselves [10]. Researchers concluded that prolonged exposure to fear positively correlated with increased anxiety [6]. The announcements of the pandemic often came with the government's policies of mandatory lockdowns to reduce the spread of COVID-19 infections. The restrictions considerably changed lifestyles and social relationships. For the first time in their lives, students experienced home quarantine. The research on increased stress and the adverse effects of at-home isolation was conducted during other pandemics and during COVID-19 [2,11]. The magnitude of the COVID-19 pandemic multiplied stress and other adverse effects and eliminated most of the social person-to-person interaction [2,12,13]. Researchers consistently show the harmful effects of social distancing and decreased social interactions during COVID-19 closures [10]. All those elements contributed to psychological dysfunction, increased stress, anxiety symptoms [14], and changes in sleeping patterns [15,16]. Other studies show a link between COVID-19-induced home isolation and changed sleep patterns [2], COVID-19 and stress, and poor academic performance [2,17].

Following announcements of public restrictions, colleges and universities rushed to implement online-only learning models [2,7-9,18,19]. Preventing academic loss and adopting digital learning allowed students to continue their education under home quarantine. Before the COVID-19 pandemic, Brooks et al. [11] analyzed the effects of home quarantine during other outbreaks. Brooks et al. hypothesize that education, when conducted under home quarantine, is a cause of increased frustration, stress, anger, and anxiety. The dominance of technology-driven college education associated with long online hours and possible internet addiction leads to a significant increase in anxiety levels [20]. The shift from in-person to digital education among university students contributed to increased anxiety prevalence [18,20]. A positive correlation between declining mental health and online learning was noted among Asian students, as observed by Islam et al. [21]. The constant fear, online presence, the enormous volume of information consumed, endless searches, and associated behaviors amplified anxiety and stress [8,22] and were a reason for the rise in anxiety [6] and were labeled as "cyberchondria" [7].

As the closures and lockdowns continued, students suffered financial hardships, causing anxiety [7,8,22,23]. College students reported adverse events such as declining family income, food and housing insecurity, and inadequate financial resources [23] to afford food, housing, and technology for effective learning. These indirect stressors disrupted the lives of low-income students more significantly and caused higher rates of stress and anxiety than those of high-income students [23]. Pandemic-generated unemployment caused working students to exhibit additional anxiety. Asian students were also reporting rising anxiety levels during the pandemic, particularly those who did not have access to resources [21], such as reliable internet, inability to purchase subscriptions, technology, or supplies, and who suffered from economic instability.

The long-term effects of the pandemic-related students' stress did not end with the first vaccination. This elevated stress trend continued for months after the initial impact [8,24-26], and even as of April 2021, 71% of Asian students were still reporting mild anxiety symptoms [26]. This finding contrasts with pre-pandemic anxiety levels recorded at about 15.7% and rising to 18.86% in March 2020 and to 32.68% in September 2020, respectively [26]. Al-Kumaim et al. [24] indicated that the pandemic had a considerable influence on students' psychological well-being, with anxiety being one of the most reported symptoms. The length of the pandemic hurt the mental health of university students. Students' anxiety levels continued at elevated levels. This observation is congruent with increased anxiety (60.8%) for students surveyed in 2021 [27]. The length of the pandemic was also detrimental to the deterioration of the mental health of the students.

Studies consistently found that female students were more at risk for increased anxiety during the pandemic. The research does not firmly establish why the female gender appears at significant risk for developing anxiety and increased stress [8,9,14-16,18,20,28-32]. This female factor could be due to the multifaceted nature of biological, psychosocial, cultural, and behavioral differences before and during the pandemic [29]. The exact calculations on how wide the gap is between male and female students' stress levels during the pandemic vary from paper to paper. The levels of pre-pandemic anxiety in female vs. male students were established in a Chinese longitudinal study showing anxiety for female students at 22%, while male students scored at 19% [29]. The study of students in Turkey [3] mid-COVID-19 demonstrates that the trend continues at almost twice the rate of anxiety, with 63% in females vs. 36% in male students. A systemic review by Liyanage et al. [4] quotes global differences in stress and anxiety for university students with mid-pandemic symptoms at 43% for females and 39% for males.

Several scholarly papers discuss the presence of a few well-established protective factors that reduce the levels of stress and anxiety experienced by students during the pandemic. The researchers zeroed in on the male gender [9,29-31,33], the presence of physical health [31], participation in exercise [34], student seniority, and urban residence [12]. Pre and mid-pandemic literature suggests that male students have shown consistently lower stress and anxiety symptoms than their female counterparts. This review shows strong mid-pandemic consistency for gender differences across many authors [3,6-9,13,28,29,31,32]. The articles reviewed do not explain why the male gender appears less affected by mental health challenges during the pandemic.

Academic stress is a part of a typical student learning cycle. Students who had senior student status experienced less stress [16,28,32]. Wang et al. [33] confirmed that increased stress and anxiety are more prevalent in junior student populations. The differences between the junior and senior students can be explained by older age, more independence, and autonomy contributing to frustrations. Undergraduate students need time to develop psychological and emotional skills to handle stress better [33].

Families of students residing in rural areas are more vulnerable to socioeconomic downturn. Rural areas experience higher rates of poverty, and students tend to be poorer than their urban colleagues who reside in urban settings [8,12,33]. Living in an urban setting with more access to resources, including high-speed internet, protects students from developing higher stress and anxiety levels [12]. Students who returned home and lived with supportive family members also reported decreased stress and anxiety levels compared to students who lived alone [8,12]. Students who lived with families had lower anxiety levels. Living with supportive parents had a protective effect on rising anxiety levels [12].

The extensive use of home confinement and prolonged self-quarantine began the COVID-19 pandemic. The disruption of students' daily regimens and increasing lack of physical health resulted in decreased motivation and academic/social involvement [31]. Xiang et al. [34] describe the relationship between anxiety levels among Chinese students who engaged in consistent physical activity during the pandemic vs. inactive students. Physically active students experienced better mental health [27,34]. Exercise stimulates endorphin production. Students who exercised regularly experienced decreased stress levels compared to non-active students [27,34]. Regular physical activity is a protective factor against increased anxiety levels. Pandemic time research showed an inverse association between resistance training and anxiety levels [34].

Together, these studies confirm the negative impact of the COVID-19 crisis on mental health, especially concerning anxiety and stress. Considering the challenges associated with the transition to adulthood, university students are particularly vulnerable to mental health problems.

Depression

Very few sources compare rates of depression pre- and post-pandemic. The studies that capture this information come from the United Arab Emirates (UAE) [28] and China [2]. They indicate the pre-pandemic level of depressive symptoms at 22% in UAE [28] and 16.9% [2] in China and post-pandemic depressive symptoms at 40% and 30.6%, respectively. The authors of both studies suggest that the rates of depression during the COVID-19 pandemic almost doubled. They link the increased rates of depression to family stress [2] and academic demands. High rates of depression at 64% among university medical students who lived with family during COVID-19 are also echoed by Serbian researchers [32]. Increased rates of depression in the student population, as compared to other age groups, before the pandemic continued the same trend post-pandemic [1].

Chinese university students who participated in the research were 18 to 24 years old [1]. This "post-adolescent" period [1] is one of the most challenging periods in life, characterized by difficulties managing emotions and inadequate capabilities and skills to manage psychological and academic needs. Research conducted during similar periods of confinement [2] shows that students, when in prolonged home isolation, are a vulnerable population to increased stress, anxiety, and, ultimately, depression. The studies on Jordanian students [2] link home confinement to COVID-19-related issues, including increased stress, anxiety, sleep and eating disturbances, feelings of loneliness, dissatisfaction with distance learning, and the presence of home quarantine as a possible precursor to the pandemic time increase in depression. Fawaz et al. [18] show a significant positive correlation between students' dissatisfaction and depressive symptoms. The analysis of the source of UK-based doctoral students' study [35] lists pre-existing cumulative stress, pre-pandemic depression, and anxiety as possible reasons for developing six times higher than the general population's depression tendencies, reporting clinical levels of depressive and anxiety symptoms at 28.3% and 79.4%, respectively. Researchers show a direct relationship between the COVID-19 home quarantine period and an increase in mental health difficulties represented by the following findings: 78% depression, 67.9% anxiety, and 58.7% stress [2].

Other studies show that during the COVID-19 pandemic, students feared and suffered academic decline [2,18,23,26,28,31]. The decrease in students' satisfaction during COVID-19 inherently influenced the increased prevalence of depressive symptoms [18,26]. Factors such as the perception of an overwhelming school workload also contributed to the increase in depressive symptoms [18,28]. Study participants felt overwhelmed by too many online tasks [18,24]. Students who are depressed are more likely to avoid classes, tests, and assignments [1], causing a slide to poor academic performance. Fawaz et al. [18] discovered an association between an abrupt shift to e-learning and an increase in perceived increase in workload that also produced depression symptoms.

Increasing depression during the COVID-19 pandemic was exacerbated among the students with feelings of loneliness and isolation [2,7,13,27]. Wang et al. [33] concluded that epidemic-related factors, such as the impact of isolation, increased anxiety, and fear of contagion, are associated with higher risk of depressive symptoms. Additional factors contributing to the increase in depressive symptoms include a family history of chronic illness [2,12].

Researchers show an increased prevalence of depressive symptoms in female students [2,29]. A Serbian study of university students [32] puts the rate of increased depression of female students at almost 2.5 times higher than men. Mirilovic et al. [32] also show more severe rates of depressive symptoms among junior first and second-year students as compared to senior students.

Despite many issues contributing to an increase in depressive symptoms, Xiang et al. [34] point to specific forms of physical activity, such as stretching and resistance training, which were negatively correlated with both anxiety and depression. Participating in house chores was negatively correlated with depression.

Quoted studies provide evidence of overall increased depressive symptoms in the university student population during the COVID-19 pandemic. The researchers point to several factors that play a role in the increased depression prevalence pre-pandemic baseline and increased mid-pandemic levels compared to the general population. This finding is a specific aspect of the mental health picture of the university student population.

Obsessive-compulsive disorder (OCD)

OCD is a chronic psychiatric disorder characterized by unwanted thoughts and repetitive behaviors. The global prevalence is at about 2.3% [36]. During the COVID-19 pandemic, government recommendations for increased handwashing efforts and other preventative measures provided a cognitive justification for excessive compulsion to take a firm root and spread [17,37,38]. Those prevention tools became a direct trigger and stressor for about 3.8% of medical students who struggled with OCD symptoms before the pandemic [36]. Medical students exhibit increased prevalence because the onset and peak of OCD happen in late teens and early adulthood. The average onset is 19-20 years old [21]. Munk et al. [38], in a German study, listed an increase in OCD symptoms from 3.6% before the pandemic to 21.4% in March 2020 in the general population. In late 2021, researchers in Iraq pointed to OCD symptoms in about 43% of surveyed university subjects [37], demonstrating a massive increase with symptoms spread as follows: unpleasant thoughts (58.1%), washing (14%), contamination scales (19.4%), and repetition of numbers (8%). These symptoms seem to affect the younger students more and the students in earlier years of study [37]. In addition, Zheng et al. [19] identified in their research that just becoming a student puts university students at 2.169 times the increased risk of having an OCD diagnosis compared to healthcare workers. Other variables as predictors for OCD in the general population may also apply to university students: being single places people at 1.836 times increased risk of having OCD, the presence of an increase in sleep latency is an independent predictor for OCD, and a history of psychiatric comorbidity. Mazhar et al. [39] observed that female medical students in junior preclinical years are more likely to suffer symptoms of OCD at increased rates in contrast to male students. Fears, depression, eating disorders, and other stress-inducing factors had a magnifying effect that contributed to increased OCD prevalence in students during the pandemic [37].

Suicidal ideation

Suicide claims over 800,000 lives every year worldwide [40], with more than half occurring before the age of 50. The estimated global mortality rate is about 16/100,000, with males at over four times the rate of females. It is the second leading cause of death among 10 to 34-year-old adults, according to the Centers for Disease Control and Prevention (CDC) [40]. Multiple authors talk about experiences of increased suicidal ideation among university students during the COVID-19 pandemic [16,30,41,42]. A study of younger students conducted before the COVID-19 pandemic by Lewinsohn et al. [43] found that female adolescents are at significantly higher risk of suicide attempts compared to male counterparts. The differences between genders diminished as participants increased with age. Gender was found to predict the lethality in suicide attempts as more males than females made attempts with high perceived lethality and medical lethality [43]. The study on Chinese university students during the COVID-19 pandemic [42] points to the male gender as well as a risk factor for increased suicidal ideation along with other factors like anxiety and depressive symptoms. Becoming a senior student decreases the risk of suicidal ideation [42].

A comparison between male and female students demonstrated that suicidal ideation is more prevalent among students with heightened depression and anxiety levels [30,41,42]. The pandemic disrupted daily structures and sleep-wake cycles. An increase in suicidal ideation was linked by AlHadi et al. [16] to students with insomnia symptoms. AlHadi et al. [16] suggest that insomnia could be the "mediator" between COVID-19 anxiety and suicide. They demonstrate a direct link between increased insomnia levels and an increase in suicidal thoughts [16]. Building on the analysis presented by Lewinsohn et al. [43], the research on Saudi students also finds junior female students have more experiences with an increased prevalence of suicidal thoughts. The theme of mental and medical health issues before the pandemic as a risk factor for developing suicidal ideation before and during the pandemic was brought up consistently as a risk factor for greater prevalence of suicidal ideation [30,40-44]. Scientists from multiple sources quoted by the CDC point out that at any age, suicidal behavior is a complex process. Experts agree that a single event does not cause suicide. It involves various risk factors, such as individual, relationships, community, and societal levels [40]. During the COVID-19 pandemic, university students were experiencing pressures and stress involving all aspects of life, increasing the possibility of the development of suicidal ideation, and advancing from ideation to suicidal attempts and suicide completion [40,42,44].

Canadian researchers [41] demonstrated that university students also remained vulnerable to the increased risk of developing suicidal ideation when additional risk factors were present: identifying as Chinese or as another ethnic minority, experiencing depression or anxiety, having a history of suicidal planning or attempts, and being overwhelmed but unable to get help [4].

The COVID-19 pandemic triggered an increase in uncertainty and fear. The elevated prevalence of suicidal ideation and other psychiatric disorders coincided with anti-pandemic measures such as lockdowns, social isolation, heightened levels of stress and psychological strain, and other factors beyond this review.

Sleeping disorders and insomnia

Most authors agree that sleeping disorders among university students have worsened during the COVID-19 pandemic, with increased stress and anxiety playing a significant role in sleep disruptions [8,10,14-16,19,20,45,46]. Students' negative perceptions during the COVID-19 pandemic were also reflected in decreased sleep quality [8,15,46]. The combination of social restrictions, physical isolation, and stress of distance learning resulted in interrupted sleep and wake cycles [8,15,20,22]. Over 52% of the students reported difficulties falling asleep and 43% reported not getting enough sleep with self-reported stress and anxiety in 78% of the student population [46], causing declining mental health [25].

Pre-COVID-19 sleep disorders increased their prevalence during the COVID-19 pandemic [15,46]. Dongol et al. analyzed data from smartwatches and surveys to measure sleep duration and light and deep sleep, and demonstrated that almost 80% of the students were found to be experiencing high-level sleeping disturbance; most significantly, about 27% of males and 33% of females had clinical insomnia [15]. These findings are in sharp contrast to insomnia presence before the pandemic at 9.5% [47]. The authors offer a list of predisposing factors that included the following: female gender, chronic health issues, being an older student, sleep disorder before the COVID-19 pandemic, increased caffeine consumption, and changes in daily routines that include sleeping patterns [15]. Dongol et al. attributed increased stress, poor sleep quality, and fear during COVID-19 to the elevated presence of clinical insomnia symptoms. On the other hand, the conclusion of the Italian study [14] discusses how the lockdowns affected the students' psycho-emotional well-being, changes in sleep patterns, and heightened insomnia rates from 15% before COVID-19 to 42% during the pandemic. Students identified different aspects associated with this difficulty: 52% reported increased problems falling asleep, 43% were not getting enough sleep, and almost 70% reported various disturbances ranging from having vivid dreams and not having a fixed sleep schedule to overwhelming stress and anxiety [14].

The fear of contracting COVID-19 has been found to correlate with heightened stress levels, clinical insomnia, and diminished sleep quality [10,15]. Pre-pandemic studies link insomnia in college students to poor academic performance [45]. Mid-pandemic research by Son et al. [10] suggested insomnia contributes to difficulties with concentration.

There has been a notable rise in the prevalence of insomnia, a decline in sleep quality, and elevated stress levels among college students, regardless of country of origin. This increased prevalence is particularly pronounced among female students [14,20], older students, and those with chronic illnesses and pre-existing sleep disorders [15,16].

Research gaps

This review addresses only college students as they are a particular population with many challenges. The scholarly papers examined the mental health difficulties, stressors, sleeping difficulties, selected psychiatric prevalence, coping methods, and potential short-term psychological impacts of the pandemic on higher education students. This review identifies opportunities for further research in this area by identifying gaps in existing literature.

More research should be conducted on the psychological influence of the coronavirus pandemic on university students with different backgrounds. The studies presented in this paper originated from the West, China, India, and a few Middle Eastern countries. It is essential to recognize how this pandemic has affected individuals from different backgrounds and how many factors may have influenced their experiences. Most studies have primarily focused on a narrow range of short-term effects. It is crucial to consider the wide-ranging consequences and lasting impact. Further research is necessary to fully comprehend the long-term mental health implications of the pandemic on university students.

Additionally, more research is needed to determine the most effective interventions/solutions for supporting university students continuously. Although most studies have primarily examined the adverse psychological effects, data are available on protective factors that can assist institutions and students. The research on the role of resilience and protective factors could provide helpful and practical information when facing COVID-19 or any other adverse event. This type of research allows the universities' governing bodies and administrators to comprehend students' mental health struggles and create effective strategies to support student well-being when facing personal, national, or global disasters such as COVID-19.

Future directions

To manage mental health needs during future outbreaks of COVID-19 or other pandemics, measurable, science-based, uniform intervention and support for university students are required. Most support should be deployed on a smaller scale and within the academic community to support individual students' needs. These services can be expanded and scaled up as needed during times of need. The literature suggests different models of examination of the interventional landscape [24]: (1) screenings and education: institutional and community-level regular screenings, broad education with outreach and follow-up, and access to tailored interventions and information for at-risk individuals. (2) Personal development: opportunities to examine and improve self-determination, self-efficacy, self-regulation, and other qualities [24]. Research out of Germany brings up a somewhat elusive concept of resilience [31] linked to a perception of control, a positive development that allows students to flourish under pressure and strengthen personal bonds. Researchers believe [31] that academic success is reinforced by the notion of "belonging" as a protective factor for mental health. Based on these findings, incorporating the teaching of resilience could become a valuable tool for university students. (3) Technical fluency: ongoing training and exposure to new technologies, digital fluency, mobile interactive learning, and intuitive design of online learning experiences [24]. (4) Environmental support: this area includes a broad category of socioeconomic factors like family support, university support, and emotional engagement [23,24]. Each category has its variables, and students' well-being depends on the sum of improvements in all four areas.

Finally, implementing a structured program of longitudinal studies to capture and measure the adequate representation of the mental health status of university students is the preferred practice in higher educational settings [4]. Taking a continuous pulse of the students' mental health through regular health screenings and hopefully accessible intervention seems like a logical solution. As those new interventions, studies, and programs are instituted, we need to measure what is changing and what is effective [25].

Recommendations

The COVID-19 pandemic caused school and university closures and changes in daily schedules, teaching methods, and other aspects of daily life. These changes dramatically impacted university students and highly affected young adults, leading to many mental health trends discussed above. The disadvantaged factors emerge among different student profiles analyzed: social isolation, female, history of comorbidities, poor, and rural background. The advantage factors were good health, male gender, resilience, supportive family and plenty of social connections, affluent urban background, and regular physical activity. The pandemic has intensified the significant increase in the number and type of disorders across the mental health spectrum and the rapid emergence of psychiatric conditions. This review finds that the COVID-19 pandemic negatively affected the mental health of university students, and the following trends have been identified: (1) decreased mental health: these psychological trends include increased stress, anxiety, self-reported decreased well-being, constant worry, etc. [1,3,4,6,9,10,12-23,26-28,30,31,33,34,46]; (2) psychiatric trends include the presence of depression, substance abuse, and increased psychiatric morbidity, including an increase in OCD occurrence as well as suicidal ideation [1,16-19,23,24,26,28,30-37,39,41,42,48]; (3) disruption of the circadian cycle trends, sleep disorders, and insomnia [10,14-16,19,20,46]; (4) the disproportional detrimental effect of COVID-19 on female students in all areas reviewed [3,6,9,13-16,20,28-31].

## Conclusions

The COVID-19 pandemic is the most significant public health threat to generational mental health. Anxiety and stress were cited as the most prevalent conditions among the student population during the COVID-19 pandemic. Higher education institutions and governments were reactive in their response to the COVID-19 pandemic. Young intellectuals suffered at increased rates from stress, anxiety, sleep-related issues, depression, and suicidal ideation. A firm action to protect the students' safety and physical, social, and mental well-being must be taken in partnership with their communities, students, and higher education institutions.
